# Reports Relating to the Sanitary Condition of the City of London

**Published:** 1855-06

**Authors:** 


### BIBLIOGRAPHICAL NOTICES.

*Reports relating to the Sanitary Condition of the City of London.* By JOHN SIMON, F. R. S., Surgeon to St. Thomas's Hospital, and Officer of Health to the City. London, 1854.

These are highly valuable and interesting sanitary reports, although local in their character. Officially addressed to the Commissioners of Sewers of the City of London, and printed originally for the use of the Corporation, nevertheless, they commend themselves to the general reader; especially to those who have given any attention to that most important yet "scandalously neglected" subject, Public Hygiene.

In this age of internal improvement, of scientific research and discovery, of changes and revolutions in daily progress, moving on, too, with a startling rapidity that in some instances would appear almost to outstrip the capabilities of our nature, we cannot comprehend the secret agency that retards the wheels of sanitary reform. It is a fact incontrovertible, that in this country, at least, there has been very little, if any, national legislation towards an effective and permanent sanitary organization. In other words, the cultivation of the science of the physical necessities of human life has received at the hands of our law-makers far less attention than any one subject which has claimed their interference. It is gratifying, however, to find here and there a mind peering out from amidst the universal indifference to those laws and regulations which are necessary to ensure the public health, and willing to devote their talents and influence to the cultivation of the sanitary science.

Prominent among such, beyond the Atlantic, may be noticed the learned author of these reports, whose indefatigable perseverance in advancing the science of Hygiene, entitles him to a distinguished position in the list of philanthropists of the nineteenth century.

In the Preface accompanying this volume of Reports, we are very modestly told, that they "lay no claim to the merit of scientific discovery." Be this as it may, they shadow forth a spirit of enquiry, and evince that philosophical familiarity with the subject, that justly entitles the author to the merit of reducing to practice certain known principles, and of adding many new facts to the domain of the science of Hygiene, for the improvement of the social, moral and physical condition of the human family; or, in his own beautiful language, he has labored "to make trite knowledge bear fruit in common application."

There are five of these reports, beginning with the year 1848 and terminating with 1853. In the fifth and last, the author sums up for this quinquennial period the death rates to the population of the city of London proper, which average about 24 *per* thousand *per annum.* A high average, when the lowest death rate *per* thousand, attained in England, for a period of seven years, has been 14. Another fact of interest which these statistics reveal, is that for the deaths exceeding five years, the rate is under 17 per thousand, while for those under this age, the rate is 85 per thousand.

After dwelling upon the abridgement of human life by preventable diseases, the *effect* of the direct operation of local and preventable *causes*, the author congratulates the Commissioners upon the success attending the public health acts of Parliament, and alludes to the sensible improvement in the sanitary condition of the city, although, he says, "the far greatest evils still remain for correction"—the deaths having been four *per cent.* fewer for equal numbers of population, than previous to the operation of these health acts. Under this head, Dr. Simon concludes by saying, "the approaching institution of your extra mural cemetery, and, I venture to hope, the translation of all slaughtering establishments to the site of your new Smithfield, will be important contributions to the regaining of the allotted duration of human life."

In all these reports, great stress is laid upon the injurious influences upon health of certain trades and occupations conducted in cities, among which, the slaughtering of animals is looked upon as a flagrant nuisance. Any occupation, the author insists, which ordinarily leaves a putrid refuse, ought not, "under any circumstances, to be located within a town;" and as to slaughtering animals in cities, he has no hesitation in pronouncing it "as both directly and indirectly prejudicial to the health of the population."

We have long entertained the opinion that any sanitary organization in a city would be incomplete, that allowed the carrying on of noxious trades, particularly those where animal matter, liable to decomposition, was in any way employed. Hence, slaughtering houses, glue and soap factories, tanneries, skin dressing, bone boiling and such like occupations, should be conducted beyond the limits of the population.

A deteriorated and unhealthy atmosphere, or an atmosphere loaded with an organic poison, must necessarily be breathed continually, wherever establishments such as have been enumerated, however well conducted, are located in the midst of a dense population. To preserve a pure and healthy state of the air in cities, all causes that exert an influence to vitiate the atmosphere should be suppressed. This opinion has been materially strengthened since reading the able reports of Dr. Simon, and we are confident that if their contents were carefully analysed and explained by competent authorities in our American cities, we should soon have legislative action, for the mitigation of many fruitful causes that exist for the prevalence of preventable diseases and deaths in cities.

House drainage and house ventilation, are also topics treated of in these essays, both of which require sanitary correction. Of the former, the experience of the author tallies with our own; viz: that imperfect house drainage is a general evil in the poorer districts of cities. It certainly prevails throughout all large cities, but not to the same extent in some districts as in others. Wherever you find a section of the city closely built upon, abounding in small houses, (with smaller yards, and in some cases, no yards at all,) and confined streets and alleys, there you have defective drainage, and with this defective drainage, you have an atmosphere congenial to the production and spread of diseases, epidemic or otherwise.

Under the head of house drainage the author enters into a very minute and efficient examination of the bad effects arising from the existence and accumulation of cesspools in cities, and says, that "it may be taken as an axiom for the purposes of sanitary improvement, that every individual cesspool is hurtful to its vicinage."

This cesspool question is one worthy of public and scientific attention. None will deny but that, at present, it is a *necessary* nuisance. In London, the evil from this source is alarming. It is bad enough in our American cities, where human ordure is deposited in partially closed, but porous sinks. But in London, according to the report of Dr. Simon, "the extreme injury which it inflicts on the health of the population, and the vital necessity of abating that injury, "has claimed the attention of his countrymen for the last ten years." In some districts he describes the numbers to be so great as resembling a cesspool city excavated beneath the city itself.

It has been a prevalent and favorite idea with us, that the present method of constructing cesspools was hurtful to health, but any improvement on the existing faulty system has been slow to work its way into popular notice.

We should take pleasure in pursuing the subject of Dr. Simon's essays still further, and to present our readers with a more elaborate and detailed notice of their truly useful contents, but the limits of our Monthly forbids other than a hasty glance.

Finally, we would that the same spirit which animates the author of the volume before us, might be infused into the minds of those who have the lead in the municipal affairs of our large cities. The good results that would thereby be effected in the improved system of sanitary enactments, by the adoption of a well regulated *health police*, would be of incalculable benefit.

This collection of reports ought to find a place in all our public libraries, and be put into the hands of the chiefs of each department of the municipal government of our principal cities.

